# More Than Meets the Kappa for Antibody Superantigen Protein L (PpL)

**DOI:** 10.3390/antib11010014

**Published:** 2022-02-11

**Authors:** Wei-Li Ling, Joshua Yi Yeo, Yuen-Ling Ng, Anil Wipat, Samuel Ken-En Gan

**Affiliations:** 1 Antibody & Product Development Laboratory, Experimental Drug Development Centre—Bioinformatics Institute, Agency for Science, Technology and Research (A*STAR), Singapore 138672, Singapore; alexling1989@gmail.com (W.-L.L.); joshy@live.com.sg (J.Y.Y.); 2 Newcastle Research and Innovation Institute (NewRIIS), Singapore 609607, Singapore; yuenling.ng@newcastle.ac.uk; 3 School of Computing, Newcastle University, Newcastle upon Tyne NE1 7RU, UK; anil.wipat@newcastle.ac.uk; 4 James Cook University, Singapore 387380, Singapore

**Keywords:** Pertuzumab, Trastuzumab, IgG1, VH families, VK families, immunoglobulin, superantigen

## Abstract

Immunoglobulin superantigens play an important role in affinity purification of antibodies and the microbiota-immune axis at mucosal areas. Based on current understanding, *Staphylococcal* Protein A (SpA), *Streptococcal* Protein G (SpG) and *Finegoldia* Protein L (PpL) are thought to only bind specific regions of human antibodies, allowing for selective purification of antibody isotypes and chains. Clinically, these superantigens are often classified as toxins and increase the virulence of the producing pathogen through unspecific interactions with immune proteins. To perform an in-depth interaction study of these three superantigens with antibodies, bio-layer interferometry (BLI) measurements of their interactions with a permutation panel of 63 IgG1 variants of Pertuzumab and Trastuzumab CDRs grafted to the six human Vκ and seven human VH region families were tested. Through this holistic and systemic analysis of IgG1 variants with various antibody regions modified, comparisons revealed novel PpL–antibody interactions influenced by other non-canonical antibody known light-chain framework regions, whereas SpA and SpG showed relatively consistent interactions. These findings have implications on PpL-based affinity antibody purification and design that can guide the engineering and understanding of PpL-based microbiota-immune effects.

## 1. Introduction

B cell superantigens bind antibodies to hyperstimulate populations of B cells independent of T cells and have been widely used for antibody affinity purification [1]. Superantigens are predominantly produced by microorganisms as a defence mechanism to escape the host immune system [2]. Notably, there are three widely used antibody superantigens also known as immunoglobulin binding proteins (IBP): Protein G (SpG) produced by groups C and G of *Streptococcal* bacteria [3], which binds to the heavy chain constant region of the IgG subtypes (IgG1–4); Protein A (SpA) produced by *Staphylococcus aureus*, which binds to the heavy chain constant region of IgG1, 2, and 4 and the variable heavy (VH) 3 framework (VH3) [4,5,6]; and Protein L (PpL) produced by *Finegoldia magna* (previously known as *Peptostreptococcus magnus*), which binds to the variable light kappa κ (Vκ) chain families 1,3,4 [7] at the framework (FWR) 1 region with influence from the other regions, e.g., distal FWRs [8]. When bound to antibodies, these superantigens can reduce the binding of the antibodies to their target antigens [9], possibly reducing avidity through steric hindrances as in the case of IgM [10]. They can also cause unwanted activation [4] with downstream effects depending on their target antibody isotype (discussed in [11,12]).

With both IgG and Vκ being the predominant isotypes in humans [13,14], superantigen proteins A, G, and L can underlie significant microbiota–immune axis interactions especially at colonised mucosal areas. Many therapeutic antibodies of the IgG and κ light chain isotypes, most notably the well-studied Pertuzumab and Trastuzumab antibodies which target Her2-positive breast cancer. 30% of invasive breast cancers and 70% of ductal carcinomas were found to overexpress Her2 antigen [15,16], making it an attractive target for anti-cancer therapy. Trastuzumab (Herceptin^®^), a humanised monoclonal IgG1 antibody with its V-region derived from rodents [17], is often coupled with Pertuzumab (Perjeta^®^) [18] in clinical therapy. Both Trastuzumab and Pertuzumab can co-localize on Her2 as they bind at different sites on Her2 [19] and have been shown to effectively treat Her2-overexpressing cancers synergistically. Using these two antibody models, we investigated unwanted interactions of such superantigens produced by commensals. In addition, implications of these antibody-superantigen interactions extend to antibody purification processes which often utilises these superantigens.

Given the common use of these three superantigens, a holistic [12,20] and systematic antibody–superantigen investigation using 63 of our previously engineered antibodies [21,22,23] involving the permutations of six human Vκ and seven human VH IgGs was performed. These recombinant antibodies were engineered based on the complementarity determining regions (CDR)-grafting of the Trastuzumab and Pertuzumab antibodies onto the human antibody FWRs on both heavy and light chains, allowing for a systematic analysis of these regions (V-region, VH, Vκ, FWRs, and CDRs of both chains, and the synergistic interactions) in superantigen engagements.

## 2. Materials and Methods

### 2.1. Recombinant Antibody Production

All Trastuzumab and Pertuzumab VH and Vκ CDR-grafted sequences used were described previously [21,22]. Briefly, the genes were sub-cloned into the pTT5 vector (Youbio, Changsha, China, Cat: VT2202) using restriction enzyme sites as previously performed [8,21,22,23,24]. The plasmids were transformed into competent *E. coli* (DH5α) strains [25] followed by plasmid extraction (Biobasic Pte Ltd, Singapore, Singapore, Cat: BS614). Transfection, production, and purification were done using HEK293E with homemade transfection agent based on linear PEI [26] (PolyScience, Singpaore, Singapore, Cat: 23966-1), followed by 14 days post-transfection supernatant harvest that were subjected to AKTA Pure system equipped with Protein G column for affinity purification and size exclusion column for collection of monomeric fractions as characterised previously [21,22].

### 2.2. Binding Affinity Quantification

Measurements of the rates of association (ka) and rates of dissociation (kd) were performed using PpL (Sartorius, Singapore, Singapore, Cat: 18-5185), SpA (Sartorius, Singapore, Cat: 18-5012), and SpG (Sartorius, Singapore, Cat: 18-18-5083) biosensors to Pertuzumab and Trastuzumab IgG1 variants described above in solution using the Octet Red96^®^ system. The Octet Acquisition v10.0 program was used to set and test the respective biosensors with the following steps for measurements: Pre-conditioning (0.2 M glycine, pH 1.52 & 10× kinetic buffer (KB), 30 s); Initial Baseline (10× KB, 60 s); Baseline (10× KB, 120 s); Association (antibody variants, 6.25 nM–100 nM, 300 s); Dissociation (10× KB, 600 s); Regeneration (0.2 M glycine, pH 1.52 & 10× kinetic buffer (KB), 30 s) as previously described [4,8,21,22,23,24,27]. The equilibrium dissociation constant (KD), ka and kd were automatically calculated by the Data Analysis v10.0 program using a 1:1 fitting model. KD measurements were deemed reliable if the response rates were above 0.1 of the respective concentrations (6.25 nM–100 nM) as recommended by the manufacturer.

## 3. Results

### 3.1. BLI Measurement of Recombinant IgG1 Variants to Protein A (SpA)

To examine the potential holistic effects of Vκ1-6 pairing with VH1-7 on antibody interactions with SpA, recombinant Pertuzumab and Trastuzumab IgG1 variants of the various pairings were studied. Notably, SpA is known to bind to the CH2 and CH3 domains of the heavy chain constant (CH).

**Figure 1:**
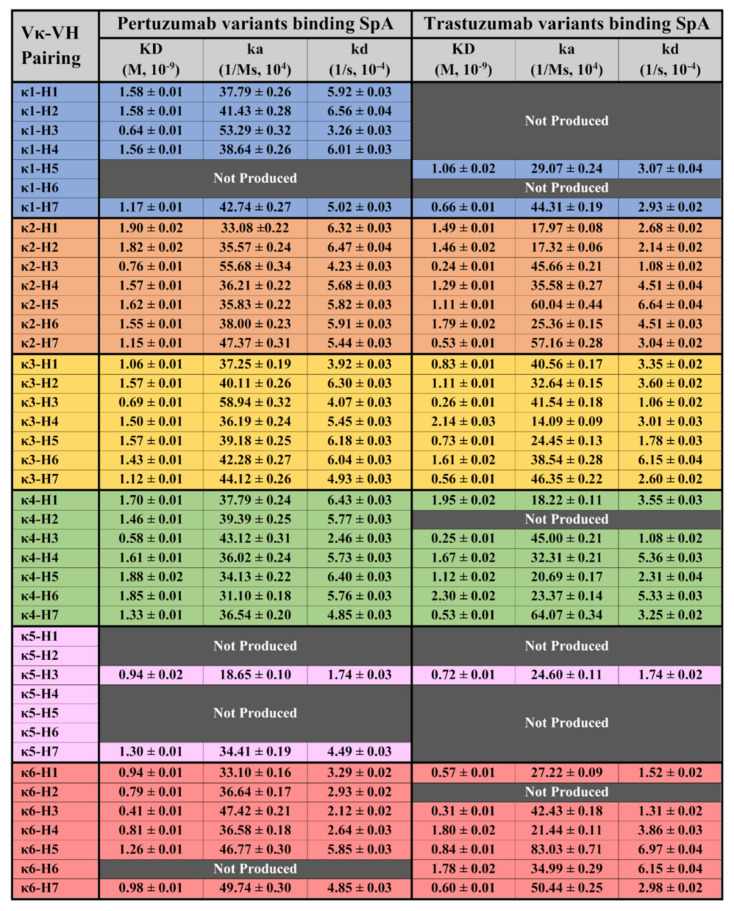
BLI measurements (KD, ka, and kd) of Pertuzumab and Trastuzumab Vκ1–6 and VH1–7variants binding to the immobilised SpA biosensor. “Not Produced” denotes insufficient antibody production for the variant despite numerous large-scale transfections. All readings were obtained from at least three antibody concentrations. The readings were the average of independent triplicates. The graphs are shown in Supplementary Figures S1–S4.

From Figure 1, the Pertuzumab and Trastuzumab IgG1 variants showed measurements bound to SpA with the KD at 0.41–1.90 × 10^−9^ M and 0.24–2.30 × 10^−9^ M, respectively (refer to Supplementary Figures S1–S4 for the graphs).

For both Pertuzumab and Trastuzumab variants binding to SpA, VH3 was noticed to have a slightly lower, albeit insignificant KD difference at the average of ~0.67 × 10^−9^ M and ~0.36 × 10^−9^ M, respectively, especially when compared to the other VH families paired with the same Vκ family. This phenomenon is attributed to the higher ka and lower kd for the VH3 variant.

### 3.2. BLI Measurement of Recombinant IgG1 Variants to Protein G (SpG)

**Figure 2:**
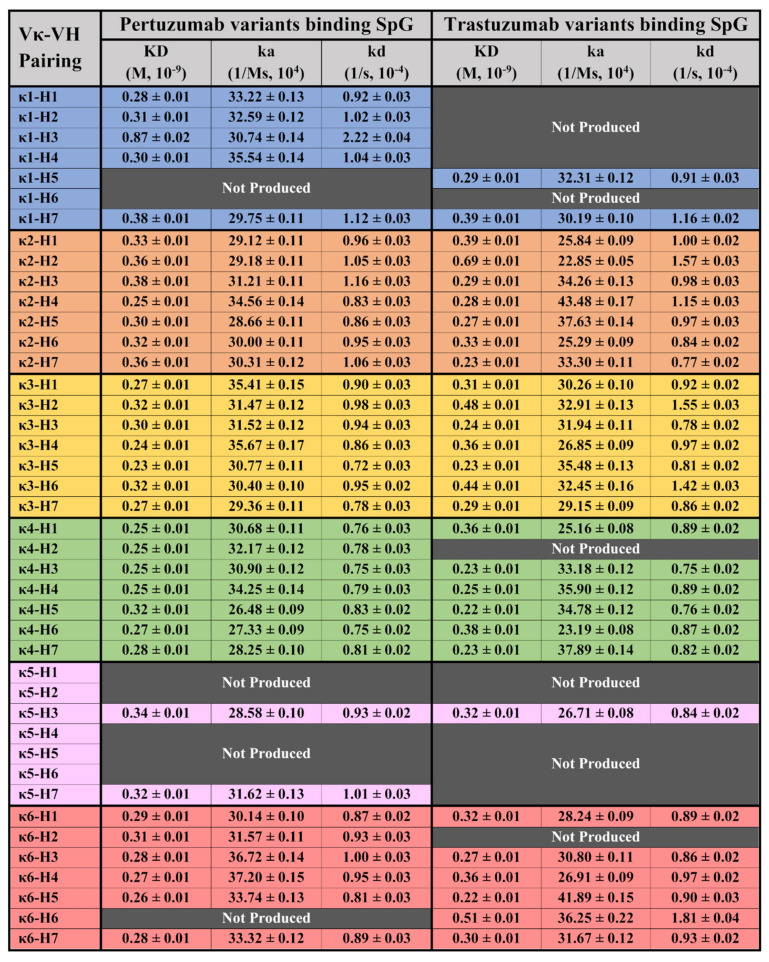
BLI measurements (KD, ka and kd) of Pertuzumab and Trastuzumab Vκ1–6 and VH1–7 variants binding to the immobilised SpG biosensor. “Not Produced” denotes insufficient antibody production for the variants despite numerous large-scale transfections. All readings were obtained from at least three antibody concentrations. The readings were the average of independent triplicates. The graphs are shown in Supplementary Figures S5–S8.

Testing the 63 recombinant Pertuzumab and Trastuzumab IgG1 variants with SpG (Figure 2), known to bind the CH2 and CH3 domains, we found high consistency of the interactions between the two Pertuzumab and Trastuzumab IgG1 variants. Apart from showing similar KDs to the SpA, SpG interactions showed narrower KD ranges of 0.23–0.87 × 10^−9^ M and 0.22–0.69 × 10^−9^ M, respectively (refer to Supplementary Figures S5–S8 for the graphs). A trend of Trastuzumab variants binding SpG better than their Pertuzumab counterparts was observed and this slight difference hints of complementarity-determining regions (CDRs) effects given that the Pertuzumab and Trastuzumab variants differed only at a few residues within the CDRs.

### 3.3. BLI Measurement of Recombinant IgG1 Variants Binding to Protein L (PpL)

**Figure 3:**
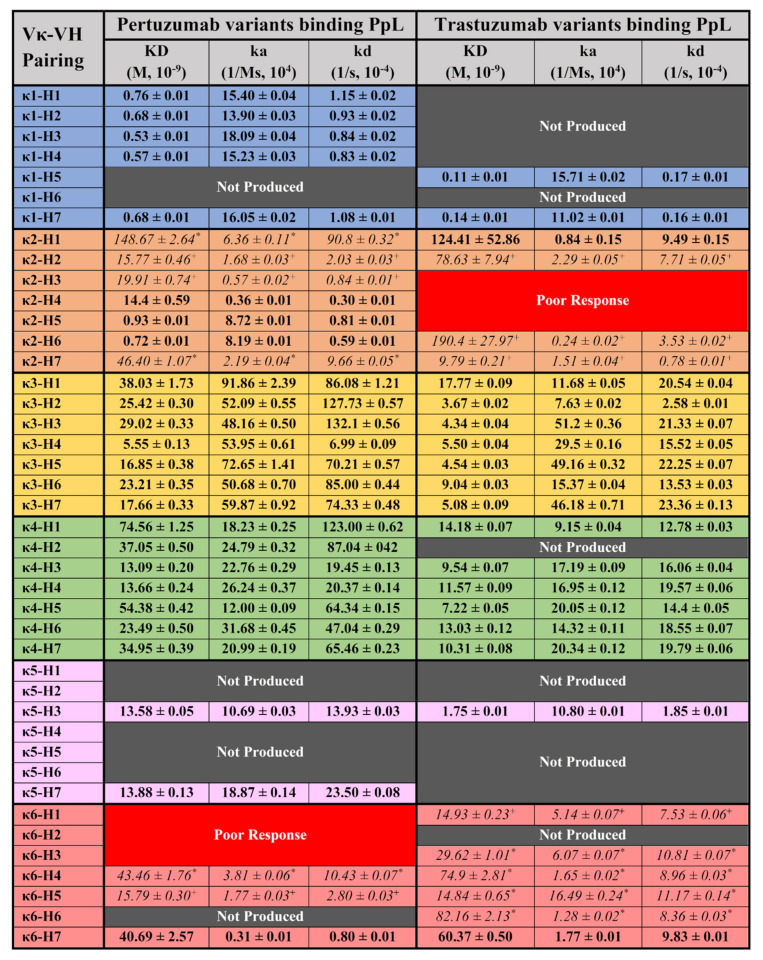
BLI measurements (KD, ka, and kd) of Pertuzumab and Trastuzumab Vκ1–6 and VH1–7 variants binding to the immobilised PpL biosensor. “Not Produced” denotes insufficient antibody production for the variants despite numerous large-scale transfections. “Poor response” indicates that the particular Vκ-VH IgG1 pairing was unable to give response rates within the detection limit across all concentrations. * denotes readings that were derived from two IgG1 concentrations. + denotes represent readings derived from only one IgG1 concentration which was deemed reliable. All other readings were obtained from at least three antibody concentrations. The readings were the average of independent triplicates. The graphs are shown in Supplementary Figures S9–S12.

Testing the same panel of antibodies on PpL (Figure 3), our systematic and holistic investigation of IgG1s to PpL showed non-canonical results of interactions with other Vκ families and a contributory role of VH-FWR and CDRs to the interaction.

As a control for expected superantigen interactions, the Pertuzumab IgG1s of Vκ1, 3, and 4 interacted with PpL, where Vκ1 showed the lowest KD range (0.53–0.76 × 10^−9^ M), followed by Vκ3 (5.55–38.03 × 10^−9^ M) and Vκ4 (13.09–74.56 × 10^−9^ M) (refer to Supplementary Figures S1–S4 for the graphs). The Vκ1 findings were consistent with our previous work [8,28,29]. The lower KDs of the Vκ3 and 4 were due to the lower dissociation rates (kd) despite the higher association rates (ka) than Vκ1. This trend was also observed for the Trastuzumab IgG1s with its Vκ1 showing the lowest KD range (0.11 and 0.14 × 10^−9^ M) followed by Vκ3 (3.67–17.77 × 10^−9^ M) and 4 (7.22–14.18 × 10^−9^ M). Note that Trastuzumab IgG1s showed lower and a narrower KD range than the Pertuzumab Vκ-VH equivalents suggesting effects from the CDRs which were what differed between the two sets of IgG1s.

Unexpectedly, certain Pertuzumab VHs paired with Vκ2, 5 and 6 exhibited interactions with PpL. Amongst these Vκ families, Pertuzumab variants Vκ5 (13.58 & 13.88 × 10^−9^ M), 6 (15.79–43.46 × 10^−9^ M) and certain Vκ2 variants (VH2–4 and 7, 14.4–46.4 × 10^−9^ M) had KDs comparable to Vκ3 and 4 variants (5.55–74.56 × 10^−9^ M). Pertuzumab Vκ2 paired with VH5 and 6 (0.93 and 0.72 × 10^−9^ M, respectively) had KDs comparable to Vκ1 (0.53–0.76 × 10^−9^ M) while Vκ2 paired with VH1 had the highest KD (poorest binding) of 148.67 × 10^−9^ M. There were also non-binding IgG1s of the Pertuzumab Vκ6 variant with VH1–3 (Poor Response) despite measurable responses when paired with VH4, 5 and 7.

Generally, the Pertuzumab trends were largely similar to the Trastuzumab IgG1s where KDs of Vκ5 (1.75 × 10^−9^ M) and 6 (14.84–82.16 × 10^−9^ M) and Vκ2 paired with VH2 & 7 (78.63 & 9.79 × 10^−9^ M, respectively) had KD values comparable to Vκ3 and 4 (3.67–14.18 × 10^−9^ M). Trastuzumab Vκ2 paired with VH1 and 6 had the highest KD (poorest interaction) at 124.41 & 190.4 × 10^−9^ M, respectively. The non-binders were Trastuzumab Vκ2 paired with VH3–5 (Poor Response) rather than in the Vκ6 family observed for Pertuzumab. These differences demonstrated a role of the CDRs and their contribution to the PpL engagement.

## 4. Discussion

We set out to investigate the interactions of antibody superantigens Protein A, G, and L systematically and holistically with the engineering of the various V-regions of IgG1 antibodies. By using CDRs of Pertuzumab and Trastuzumab grafted onto Vκ1-6 and VH1-7 FWRs, measurements to the superantigens showed no major differences for SpA between Pertuzumab and Trastuzumab IgG1 variants (Figure 1). This was expected given that SpA bound IgG1s predominantly at the CH2-CH3 domains [30] with some contributions from the VH3 framework [4] that is also observed here to a lesser extent where the Vκ chains paired with VH3 showed a slightly lower KD (better) measurement compared to the other variants. Yet, this difference is notably less pronounced compared to our previous work on IgEs with the same Vκs-VH [4], where the VH3-CDR2 S58 residue had a more significant role in SpA binding for IgEs possibly due to its structural contribution and the Cε region.

With respect to SpG interactions, no notable differences in KDs (Figure 2) were observed among the 63 IgG1 variants. Admittedly, there are other non-specific purification methods such as the Melon™ gel which could avoid biases introduced by Protein G affinity purification. However, given that our culture medium contains low Ig FBS, utilising such a method could result in potential cross contamination of unspecific bovine antibodies (such as IgA and IgM, even with an additional size exclusion step). We thus utilised Protein G purification for consistency with our previous work [21] in which we performed similar analyses. Additionally, SpA interactions were found to be highly consistent (Figure 1) despite binding to a different site from SpG [31], reaffirming that there is no rationale to suspect interferences from using SpG purification on the Octet^®^ measurements. Nonetheless, future studies can utilise other immunoaffinity resins or the Melon gel if they wish to confirm these findings. Notably, our size exclusion purification of monomeric IgG fractions does not show residual binding of SpG to interfere with our subsequent measurements and that affinity purifications do not rely on the small changes in ka and kd that are measured here, but on the general binding between the antibody and SpG. In fact, we found a narrow KD range between the IgGs and SpG that is likely due to the lack of interference from the V-regions present for SpA. While SpG was previously reported [32] to bind to IgG1 better than SpA, this trend was more pronounced in our study.

Measurements of PpL interactions to Pertuzumab and Trastuzumab variants expectedly showed that VHs paired with Vκ1, 3 and 4 exhibits interactions as previously reported [7,8]. Surprisingly, we found non-canonical interaction of PpL with Vκ2 previously determined to not bind PpL [7] while there were no known reports of Vκ5 & 6 interactions at the time of writing. Producing these light chains as secreted dimers, we also affirmed that Vκ2, 5 and 6 light chains did not interact with PpL on the same BLI experiments (Supplementary Figure S13). Notable binders to PpL are: Pertuzumab Vκ2–VH1–7; Vκ5–VH3 & 7; Vκ6–VH4, 5 & 6; Trastuzumab Vκ2–VH1, 2, 5 & 6; Vκ5–VH3; Vκ6–VH1, 3–7 (Figure 3).

Although the novel Vκ IgG1s bound PpL showed comparable KDs, it should be noted that the KDs were calculated from one (denoted as “+” in Figure 3) or two (denoted as “*”) antibody concentrations, generally from the highest concentrations (100 nM and below) of the Ig variant. The notable exceptions were that of Pertuzumab Vκ2–VH4–6, Vκ5–VH3 & 7, Vκ6–VH6, Trastuzumab Vκ2–VH1, Vκ5–VH3, Vκ6–VH7 with KDs calculated from at least three concentrations. Interestingly, two variants: Pertuzumab Vκ2–VH5 & 6 showed KDs comparable to Vκ1–VHs values.

The unexpected IgG1 variants interacting with PpL suggested a combined VH-Vκ induced binding site to PpL that may be similar to the non-canonical binding of IgEs to nickel [4] in our previous work using the same V-regions. In fact, the IgG1 variants were validated with the expected interactions to SpA and SpG in this study to complement our previous work on their interactions with Fcγ2A and Her2 [21]. Given the lack of interactions between Vκ 2, 5, and 6 with PpL and consistency between the Trastuzumab and Pertuzumab variants, where Pertuzumab Vκ6–VH1–3 and Trastuzumab Vκ2–VH3–5 were non-binders (labelled as “Poor Response” pairs in Figure 3), PpL interaction involved more than V-region pairings alone.

With the differences between the Pertuzumab and Trastuzumab which share very similar V-regions, our findings further demonstrate the need for a design thinking [12] approach involving holistic antibody investigations approach [20]. Such an approach allowed detailed investigations for unexpected interactions between the antibodies with other proteins that can have notable immune effects, as was with our unexpected findings of IgAs binding to SpG [9]. With relevance to the development of therapeutics where a personalised antibody approach may be beneficial to avoid unwanted side effects, such interactions may also be engineered for purification purposes.

## Data Availability

Data is available upon request.

## Supplementary Materials

The following supporting information can be downloaded at: https://www.mdpi.com/article/10.3390/antib11010014/s1, Figure S1: BLI measurement of Pertuzumab and Trastuzumab Vκ1–6 binding to immobilised PpL biosensor, Figure S2: Binding graph of Pertuzumab variants (PVK3 PVH7 to PVK6 PVH7) to Protein A, Figure S3: Binding graph of Trastuzumab variants (HVK1 HVH5 to HVK3 HVH5) to Protein A, Figure S4: Binding graph of Trastuzumab variants (HVK3 HVH7 to HVK6 HVH7) to Protein A, Figure S5: Binding graph of Pertuzumab variants (PVK1 PVH1 to PVK3 PVH5) to Protein G, Figure S6: Binding graph of Pertuzumab variants (PVK3 PVH7 to PVK6 PVH7) to Protein G, Figure S7: Binding graph of Trastuzumab variants (HVK1 HVH5 to HVK3 HVH5) to Protein G, Figure S8: Binding graph of Trastuzumab variants (HVK3 HVH7 to HVK6 HVH7) to Protein G, Figure S9: Binding graph of Pertuzumab variants (PVK1 PVH1 to PVK3 PVH5) to Protein L, Figure S10: Binding graph of Pertuzumab variants (PVK3 PVH7 to PVK6 PVH7) to Protein L, Figure S11: Binding graph of Trastuzumab variants (HVK1 HVH5 to HVK3 HVH5) to Protein L, Figure S12: Binding graph of Trastuzumab variants (HVK3 HVH7 to HVK6 HVH7) to Protein L, Figure S13: BLI measurement of Pertuzumab and Trastuzumab Vκ1-6 binding to immobilized PpL biosensor.
