# Supplementary material for: More Than Meets the Kappa for Antibody Superantigen Protein L (PpL)
Source: Antibodies (Basel). 2022 Feb 11;11(1):14. doi: 10.3390/antib11010014 (PMC8883962; doi:10.3390/antib11010014)
Supplement: Supplementary file 1 [file antibodies-11-00014-s001.zip › antibodies-1565394-supplementary.pdf]

Supplementary materials

Measured SpA BLI interactions with the Pertuzumab and Trastuzumab variants

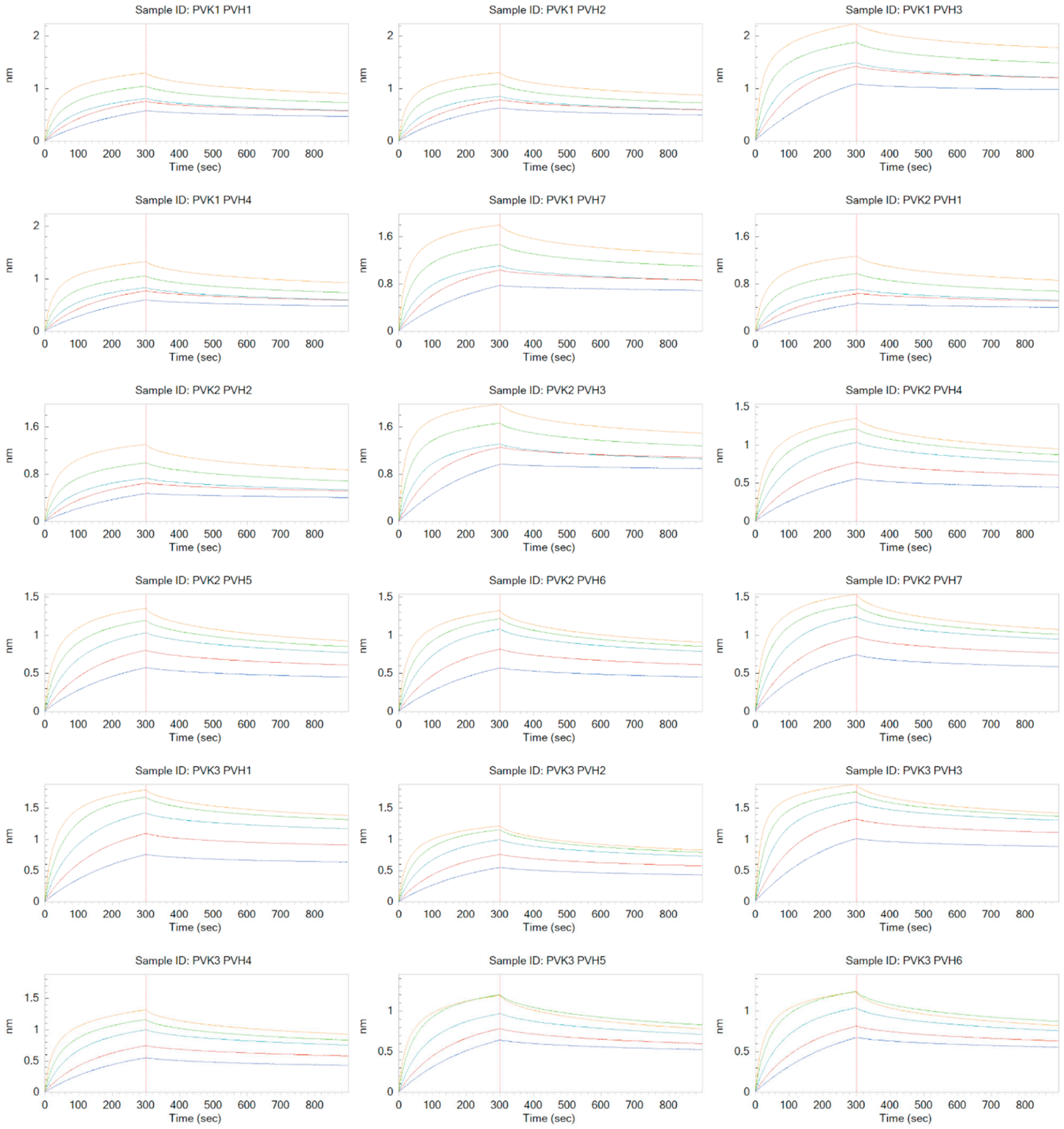

Figure S1: Binding graph of Pertuzumab variants (PVK1 PVH1 to PVK3 PVH5) to Protein A.

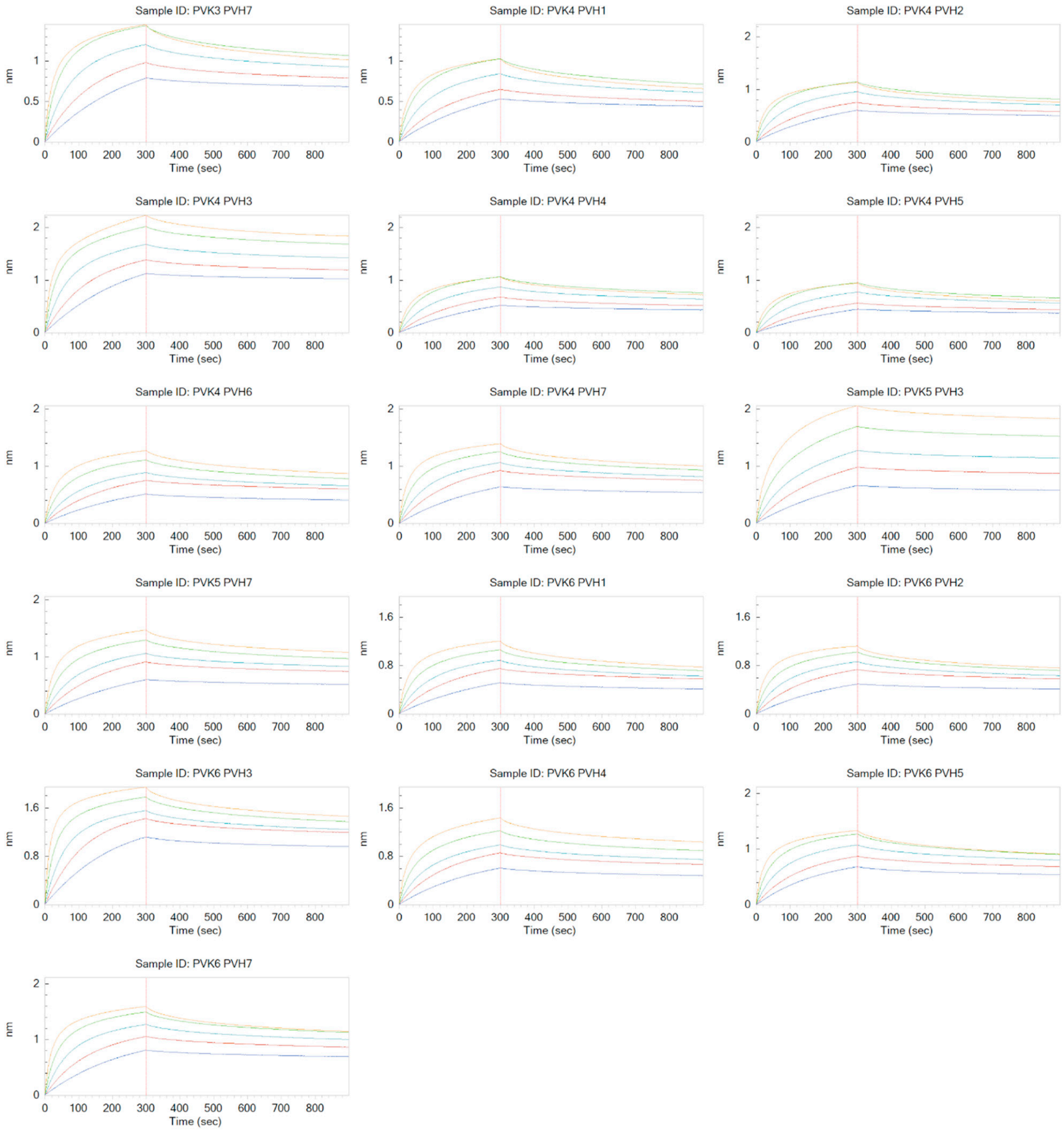

Figure S2: Binding graph of Pertuzumab variants (PVK3 PVH7 to PVK6 PVH7) to Protein A.

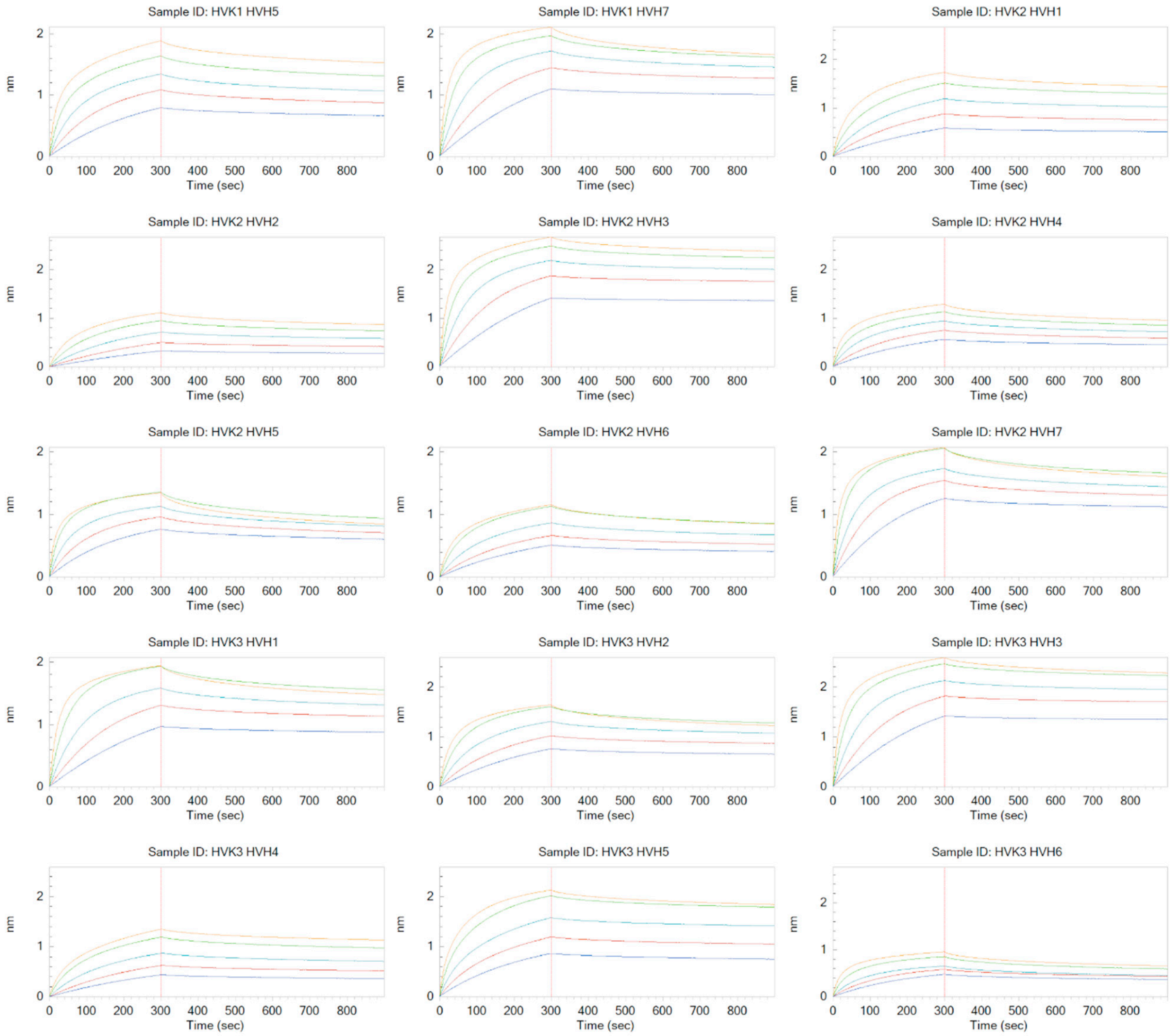

Figure S3: Binding graph of Trastuzumab variants (HVK1 HVH5 to HVK3 HVH5) to Protein A.

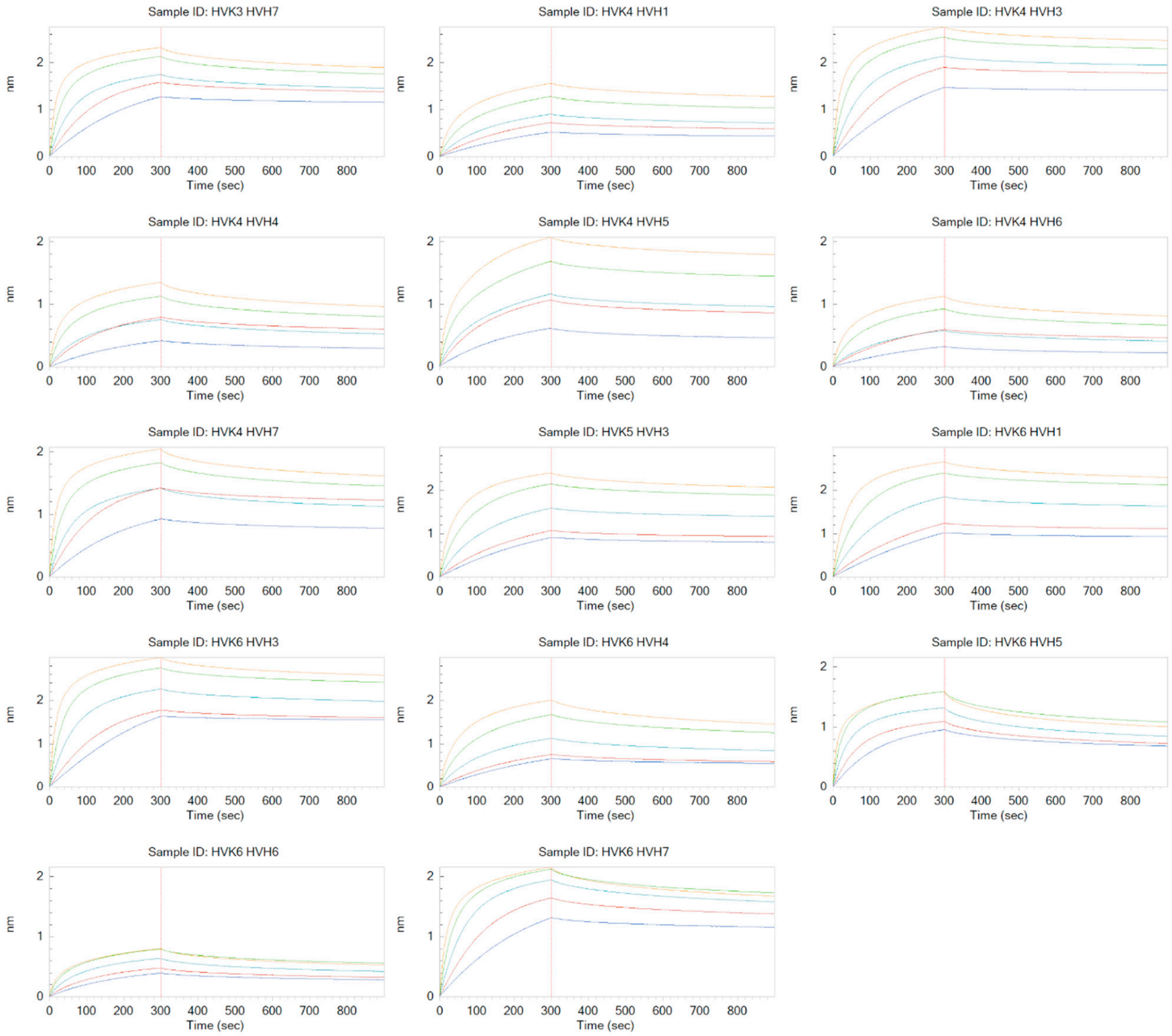

Figure S4: Binding graph of Trastuzumab variants (HVK3 HVH7 to HVK6 HVH7) to Protein A.

Measured SpG BLI interactions with the Pertuzumab and Trastuzumab variants

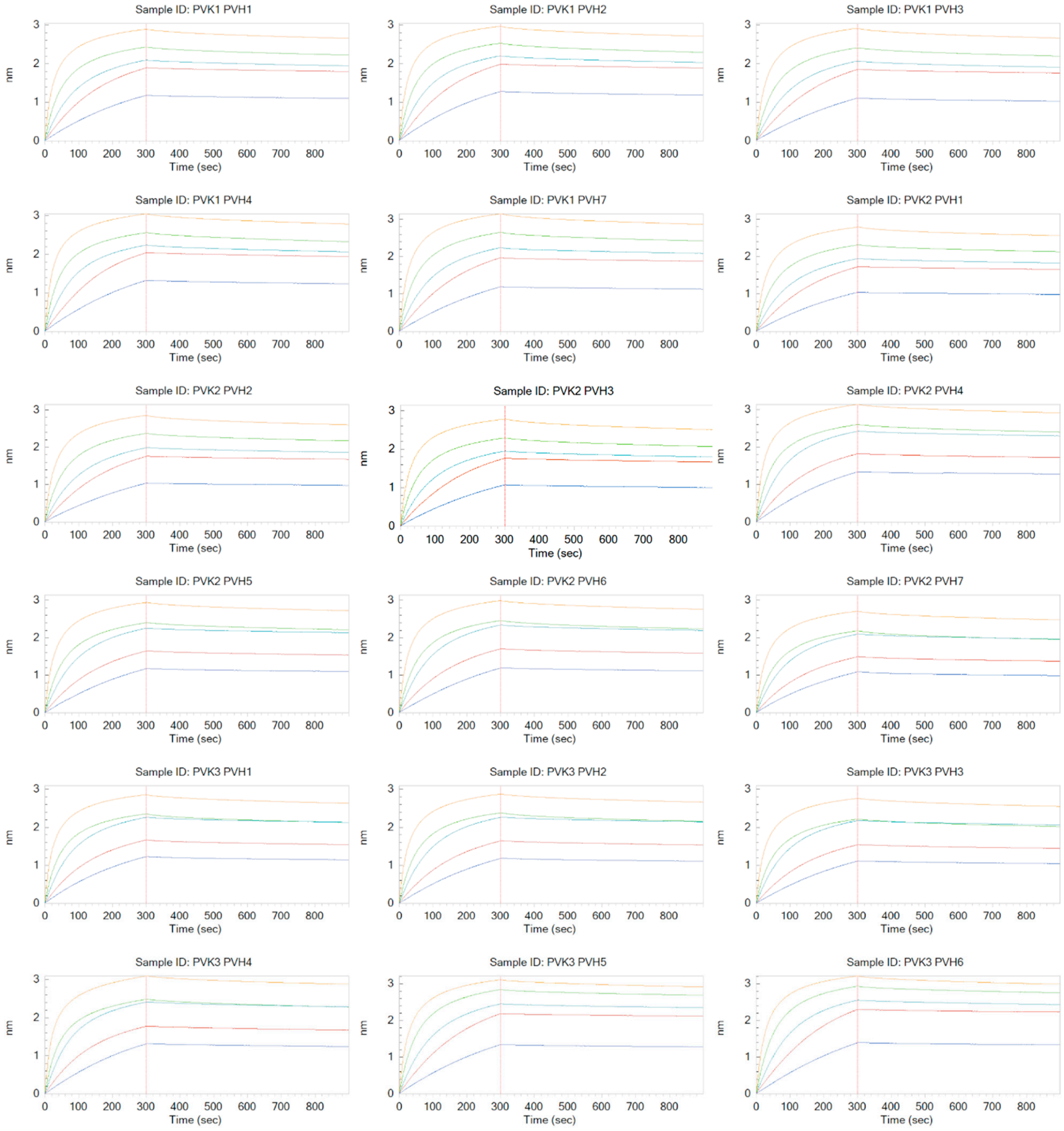

Figure S5: Binding graph of Pertuzumab variants (PVK1 PVH1 to PVK3 PVH5) to Protein G.

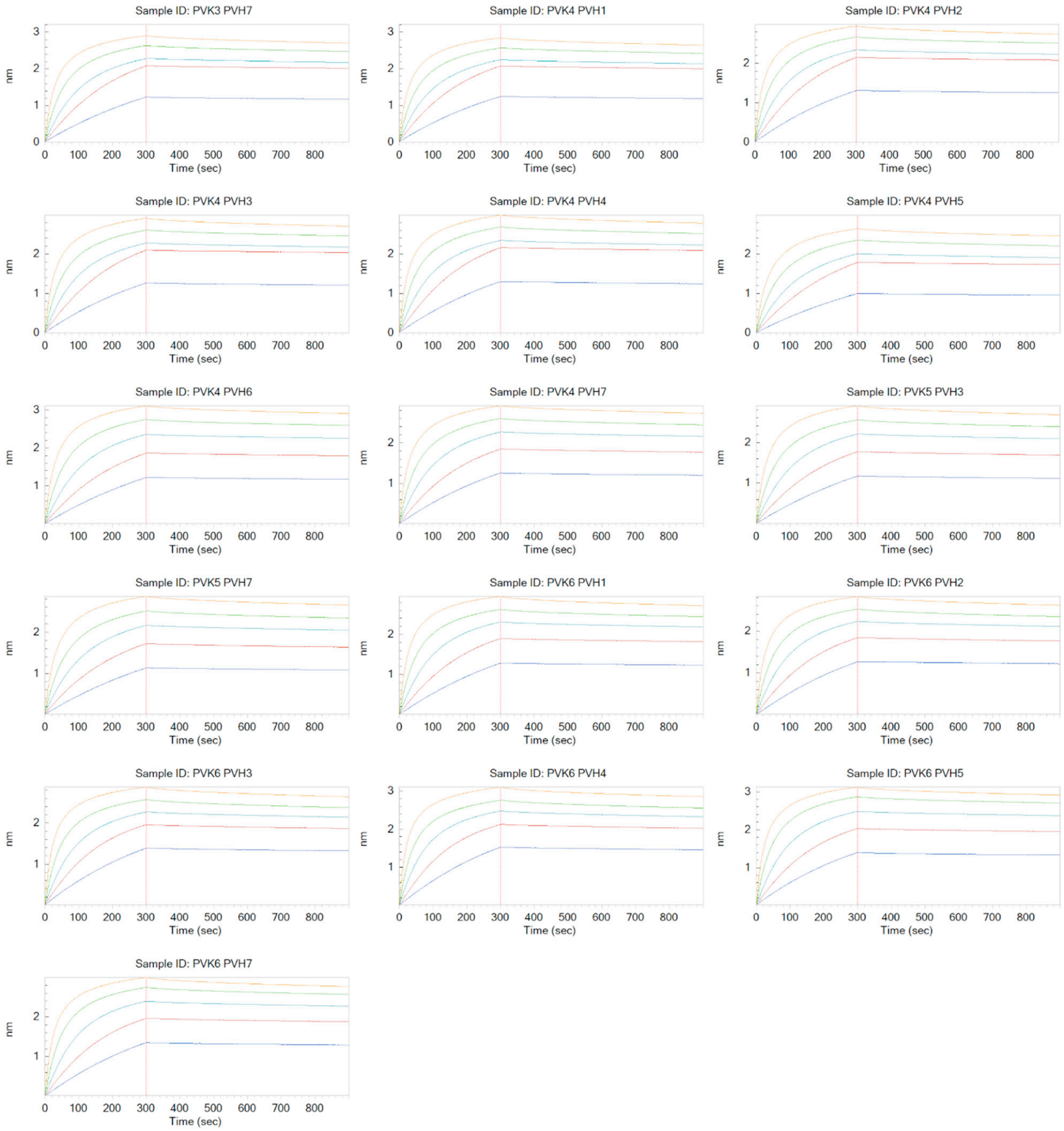

Figure S6: Binding graph of Pertuzumab variants (PVK3 PVH7 to PVK6 PVH7) to Protein G.

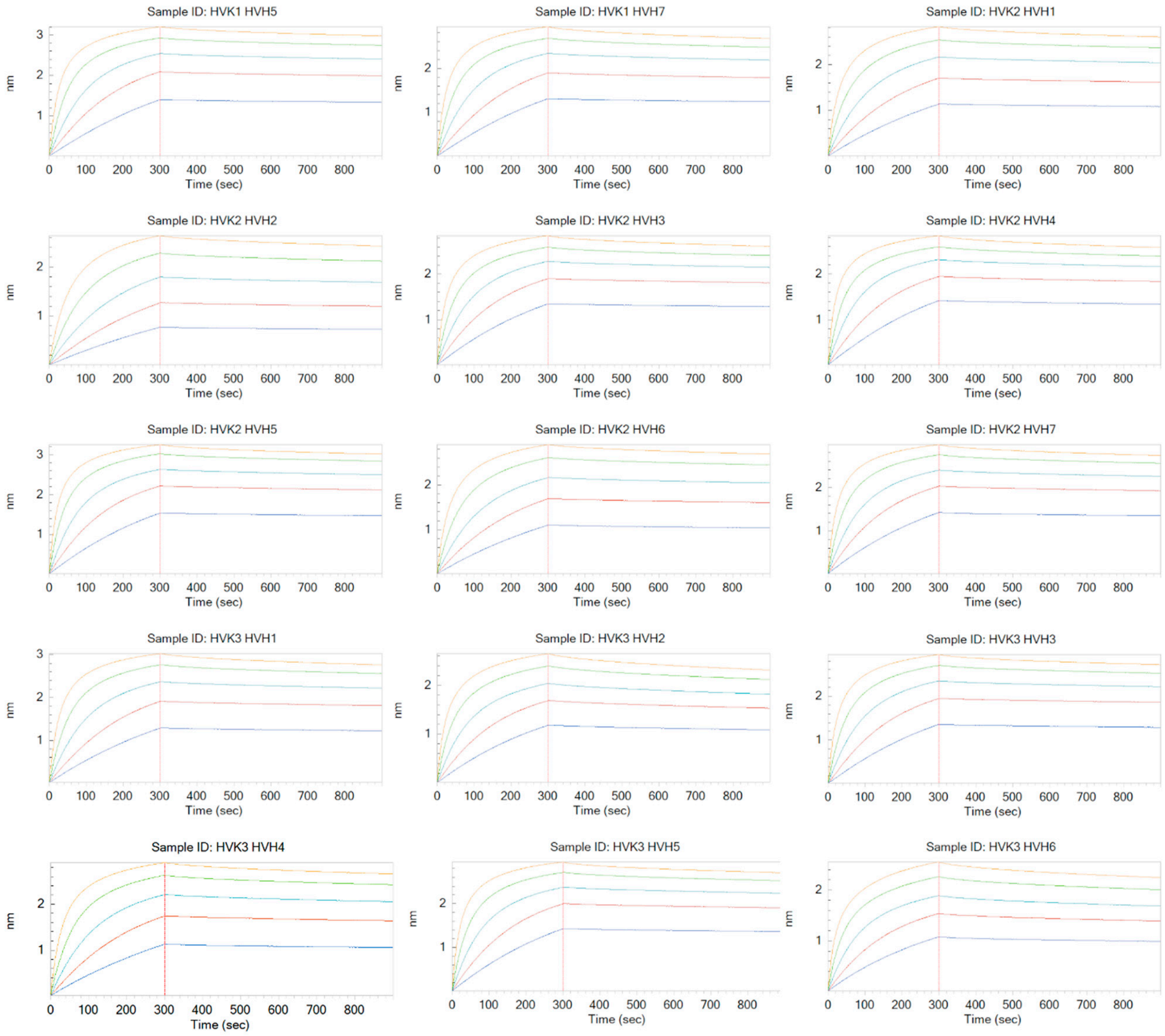

Figure S7: Binding graph of Trastuzumab variants (HVK1 HVH5 to HVK3 HVH5) to Protein G.

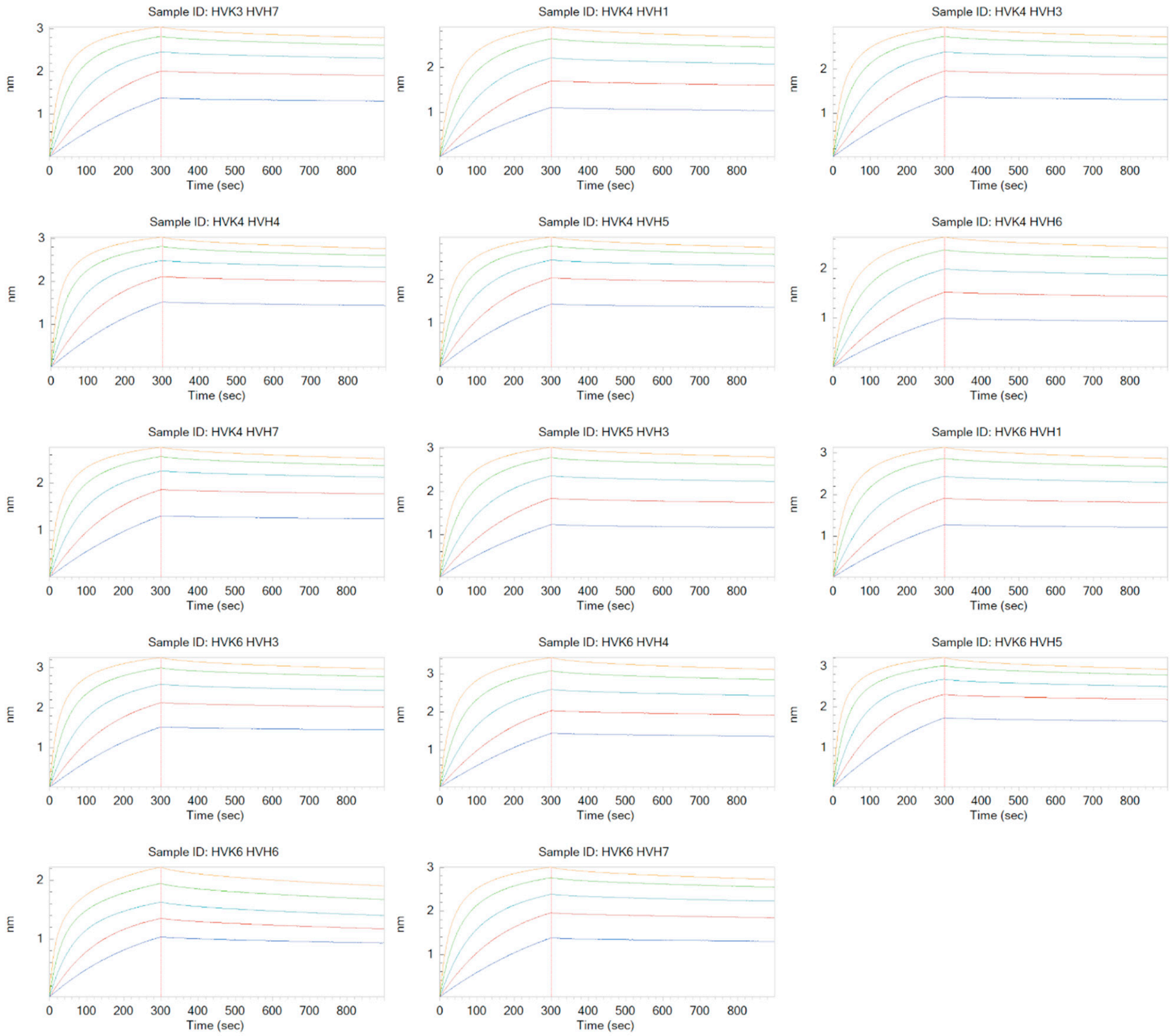

Figure S8: Binding graph of Trastuzumab variants (HVK3 HVH7 to HVK6 HVH7) to Protein G.

Measured PpL BLI interactions with the Pertuzumab and Trastuzumab variants

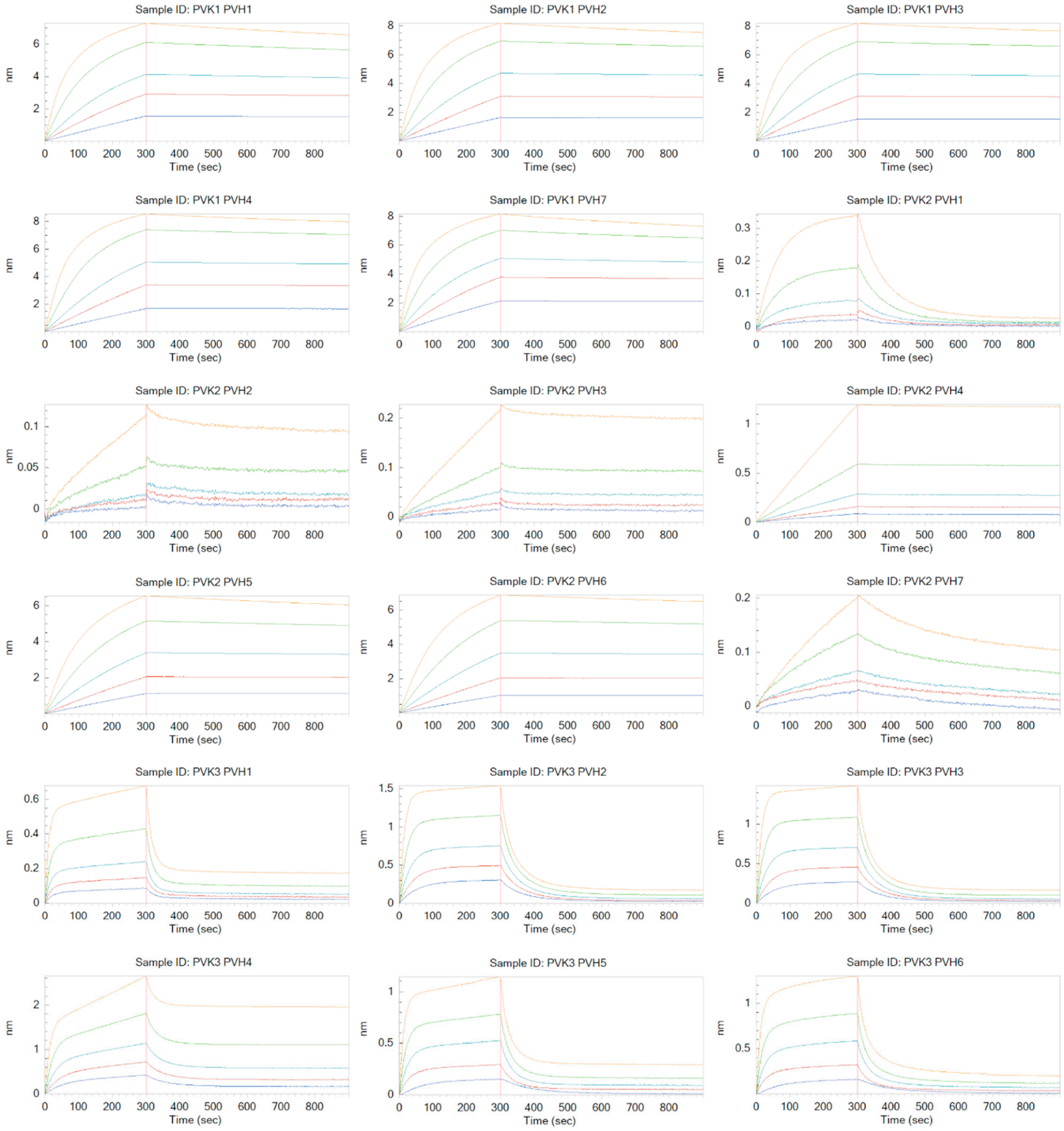

Figure S9: Binding graph of Pertuzumab variants (PVK1 PVH1 to PVK3 PVH5) to Protein L.

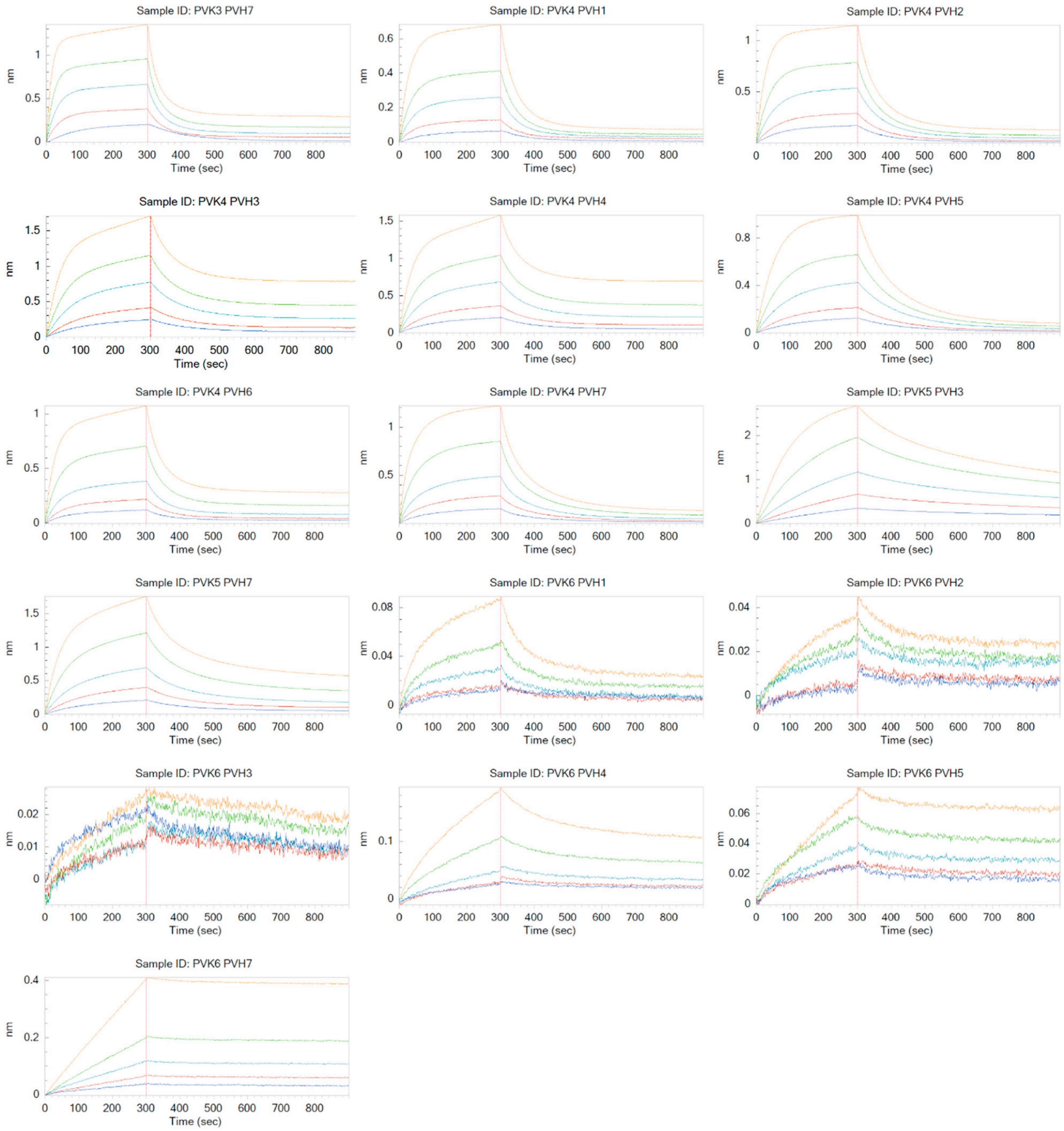

Figure S10: Binding graph of Pertuzumab variants (PVK3 PVH7 to PVK6 PVH7) to Protein L.

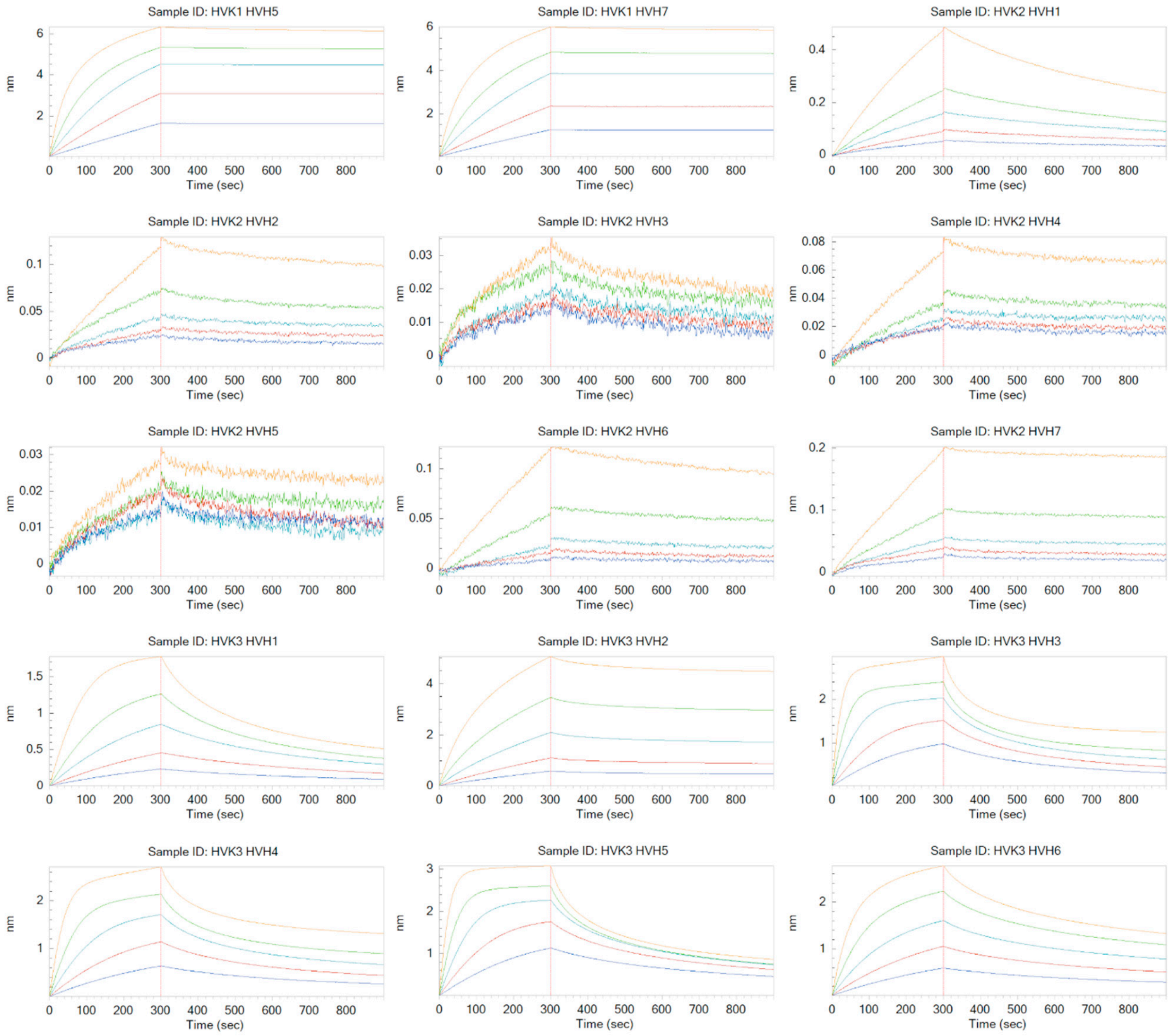

Figure S11: Binding graph of Trastuzumab variants (HVK1 HVH5 to HVK3 HVH5) to Protein L.

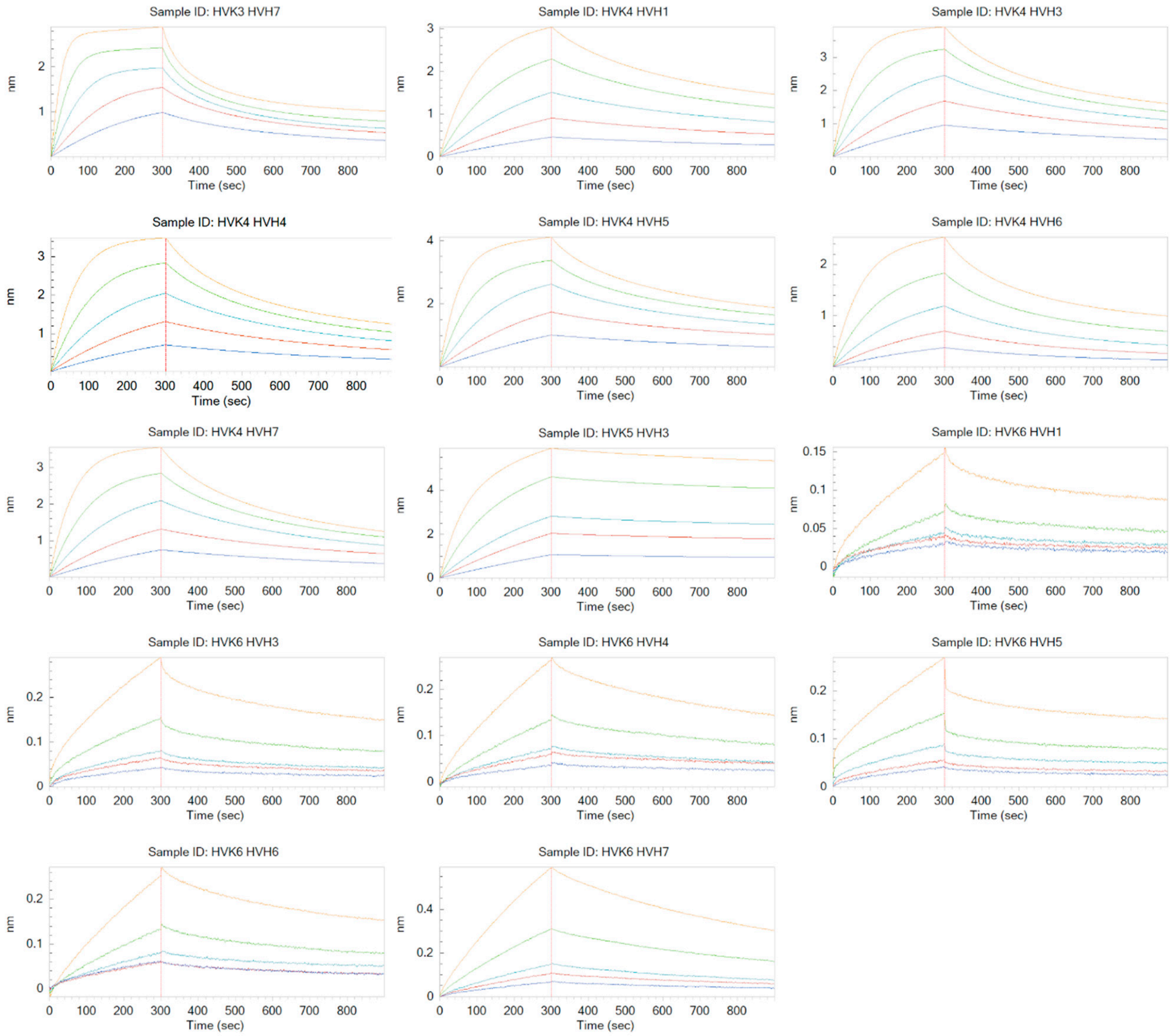

Figure S12: Binding graph of Trastuzumab variants (HVK3 HVH7 to HVK6 HVH7) to Protein L.

BLI Measurements of Pertuzumab and Trastuzumab κ-chains to PpL

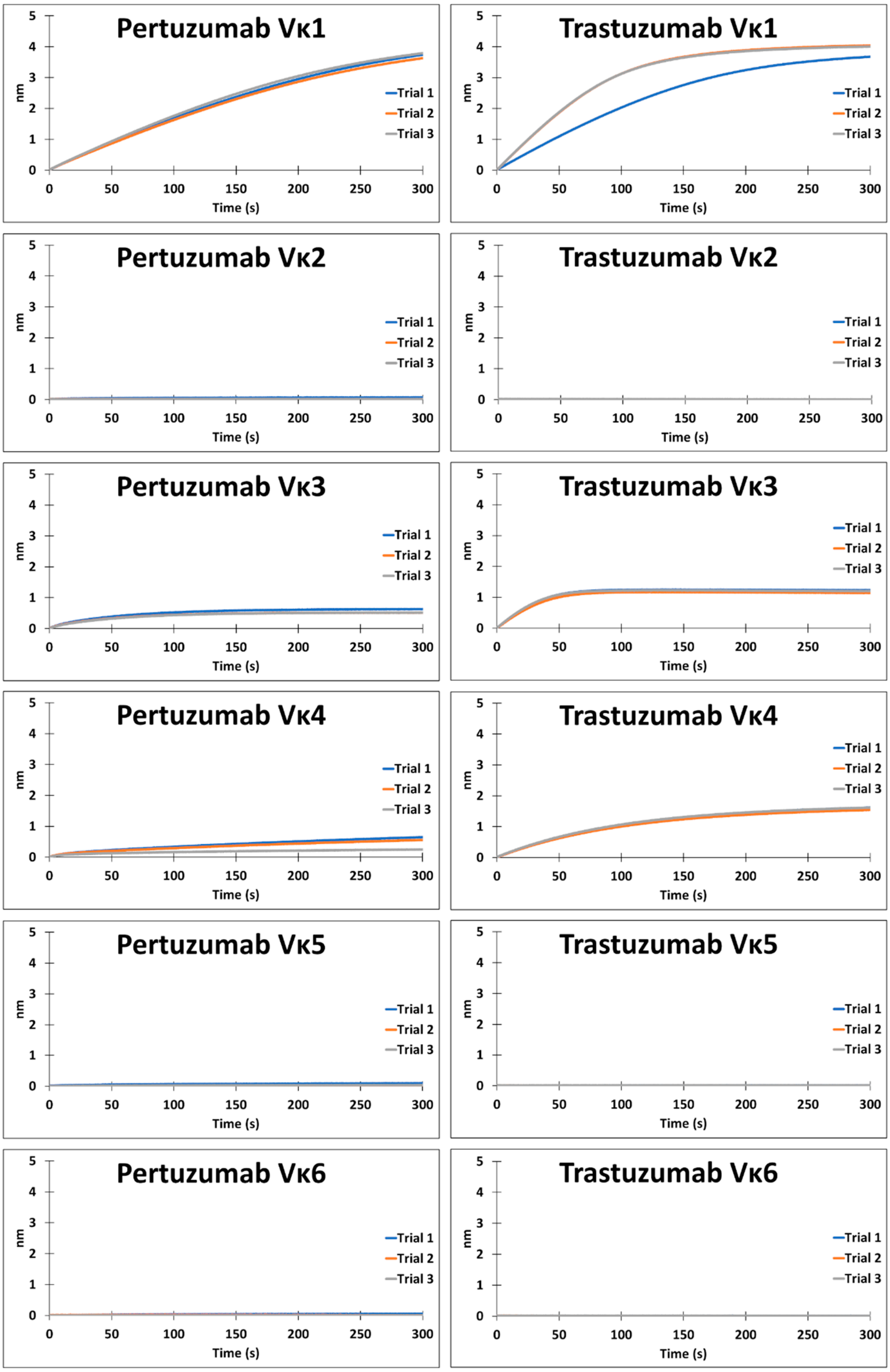

Figure S13: BLI measurement of Pertuzumab and Trastuzumab Vκ1-6 binding to immobilized PpL biosensor.
